# Characterization of two enzymes from *Psychrobacter cryohalolentis* that are required for the biosynthesis of an unusual diacetamido-d-sugar

**DOI:** 10.1016/j.jbc.2021.100463

**Published:** 2021-02-25

**Authors:** Michael P. Linehan, James B. Thoden, Hazel M. Holden

**Affiliations:** Department of Biochemistry, University of Wisconsin, Madison, Wisconsin, USA

**Keywords:** acetyl-CoA, *N*-acetyltransferase, aminotransferase, carbohydrate, enzyme kinetics, lipopolysaccharide, pyridoxal 5'-phosphate, structure–function, tertiary structure, X-ray crystallography, ATP, adenosine triphosphate, CoA, Coenzyme A, CTP, cytidine triphosphate, DEAE, diethylaminoethyl, DTT, dithiothreitol, HEPES, *N*-2-hydroxyethylpiperazine-*N*′-2-ethanesulfonic acid, HEPPS, *N*-2-hydroxyethylpiperazine-*N*′-3-propanesulfonic acid, GlcNAc, *N*-acetylglucosamine, HPLC, high-performance liquid chromatography, Ni-NTA, nickel-nitrilotriacetic acid, PLP, pyridoxal 5'-phosphate, rTEV, recombinant Tobacco Etch Virus, Tris, *tris*-(hydroxymethyl)aminomethane, UDP, uridine diphosphate, UTP, uridine triphosphate, UDP-GlcNAc, UDP-*N*-acetylglucosamine

## Abstract

*Psychrobacter cryohalolentis* strain K5^T^ is a Gram-negative organism first isolated in 2006. It has a complex O-antigen that contains, in addition to l-rhamnose and d-galactose, two diacetamido- and a triacetamido-sugar. The biochemical pathways for the production of these unusual sugars are presently unknown. Utilizing the published genome sequence of the organism, we hypothesized that the genes 0612, 0638, and 0637 encode for a 4,6-dehydratase, an aminotransferase, and an *N*-acetyltransferase, respectively, which would be required for the biosynthesis of one of the diacetamido-sugars, 2,4-diacetamido-2,4,6-trideoxy-d-glucose, starting from UDP-*N*-acetylglucosamine. Here we present functional and structural data on the proteins encoded by the 0638 and 0637 genes. The kinetic properties of these enzymes were investigated by a discontinuous HPLC assay. An X-ray crystallographic structure of 0638, determined in its external aldimine form to 1.3 Å resolution, demonstrated the manner in which the UDP ligand is positioned into the active site. It is strikingly different from that previously observed for PglE from *Campylobacter jejuni*, which functions on the same substrate. Four X-ray crystallographic structures were also determined for 0637 in various complexed states at resolutions between 1.3 and 1.55 Å. Remarkably, a tetrahedral intermediate mimicking the presumed transition state was trapped in one of the complexes. The data presented herein confirm the hypothesized functions of these enzymes and provide new insight into an unusual sugar biosynthetic pathway in Gram-negative bacteria. We also describe an efficient method for acetyl-CoA synthesis that allowed us to overcome its prohibitive cost for this analysis.

The bacterium *Psychrobacter cryohalolentis* K5^T^ is a Gram-negative organism that was first isolated in 2006 from the permafrost in the Kolyma lowland, Siberia, Russia (1). It belongs to the genus *Psychrobacter*, which includes aerobic organisms that range from psychrophilic to psychrotolerant. These organisms are also halotolerant. Strikingly, the growth temperature range for *P. cryohalolentis* K5^T^ is between –10 and 30^o^C (1).

The name *Psychrobacter*, itself, was first proposed in 1986 with the isolation of *Psychrobacter immobilis* (2). This Gram-negative food-spoilage bacterium is often associated with fish, processed meats, and poultry products (2, 3, 4). Since 1986, additional strains of *Psychrobacter* have been discovered as more research is directed toward understanding the ecology of polar and marine habitats. Although still considered opportunistic pathogens, some related strains of *P. cryohalolentis* have been isolated, for example, from human blood, cerebrospinal fluid, urine, and cutaneous sources (5). A number of clinical cases have been reported, including the recent infection of a 26-year-old man after suffering a hand laceration while crabbing in Puget Sound (6).

Recently, the structure of the O-antigen from *P. cryohalolentis* K5^T^ was reported and is displayed in Figure 1 (7). In addition to the unusual sugar, 2,3,4-triacetamido-2,3,4-trideoxy-l-arabinose, the O-antigen also contains two diacetamido d-sugars, l-rhamnose, and d-galactose. To date, there are no published reports on the structures and functions of the enzymes involved in the bioproduction of these amino sugars in *P. cryohalolentis* K5^T^. Given our long-standing interest in unusual sugar biosynthesis, we conducted an analysis of the *P. cryohalolentis* K5^T^ genome sequence. Starting from UDP-*N*-acetylglucosamine (UDP-GlcNAc), we hypothesized that genes 0612, 0638, and 0637 in *P. cryohalolentis* K5^T^ encode for a 4,6-dehydratase, a pyridoxal 5'-phosphate (PLP)-dependent aminotransferase, and an *N*-acetyltransferase, which would be required for the biosynthesis of 2,4-diacetamido-2,4,6-trideoxy-d-glucose as indicated in Figure 2. For the analysis presented here, the three above-mentioned genes were cloned, and the proteins encoded by them were overexpressed and purified. In the case of the 0612-encoded protein, the gene sequence suggested that it most likely contained a transmembrane region and a linker domain as previously observed for PglF from *Campylobacter jejuni* (8). Consequently, truncation mutant variants were prepared as was done for PlgF in order to obtain soluble protein. Whereas it was possible to achieve reasonable protein expression of the 0612-encoded protein, once it was purified it behaved poorly in terms of solubility. All attempts to stabilize the protein were unsuccessful. However, the proteins encoded by 0638 and 0637 were stable.

**Figure 1:**
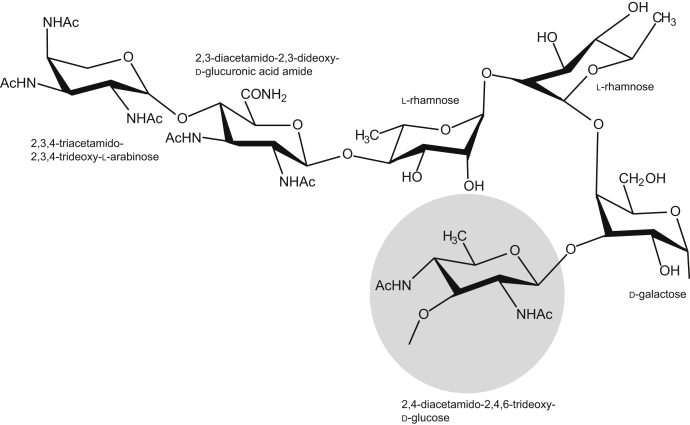
**Structure of the O-antigen from *P. cryohalolentis* K5**^**T**^.

**Figure 2:**
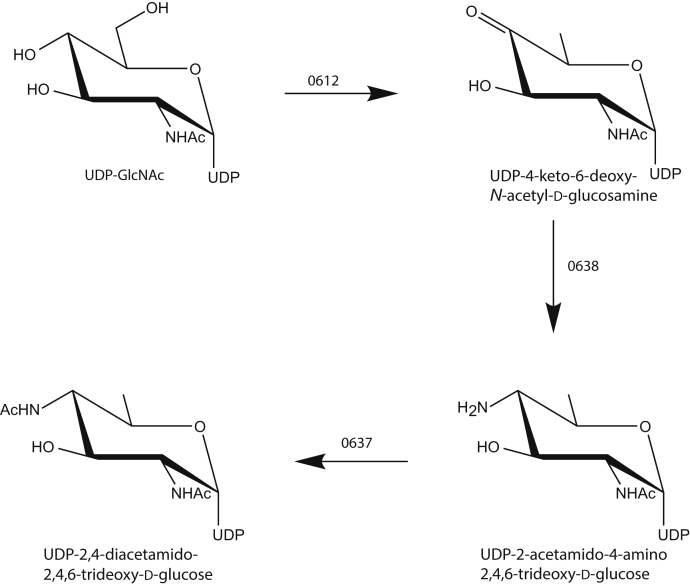
**Predicted pathway for the biosynthesis of UDP-2,4-diacetamido-2,4,6-trideoxy-d-glucose**.

In this report we present functional and structural data that demonstrate the proteins encoded by the 0638 and 0637 genes do indeed correspond to an aminotransferase (hereafter referred to as *Pcryo*_0638) and an *N*-acetyltransferase (*Pcryo*_0637). Importantly, the four high-resolution structures of the *N*-acetyltransferase reported provide further insight into this class of CoA-dependent left-handed β-helix superfamily members. Indeed, one of the structures is that of a tetrahedral intermediate.

We also provide in Experimental procedures detailed methods for the efficient synthesis of both UDP-GlcNAc and acetyl-CoA, which allowed us to overcome their prohibitive costs. These methods will prove invaluable to other researchers investigating UDP-GlcNAc and acetyl-CoA dependent enzymes.

## Results

### Synthesis of UDP-GlcNAc and acetyl-CoA.

Functional and structural investigations on enzymes involved in unusual sugar biosynthesis are often hampered because many of the ligands are not commercially available or are too costly for the quantities required for kinetic assays and X-ray crystallographic analyses.

In order to conduct the biochemical investigations described here on both *Pcryo*-0638 and *Pcryo*-0637, it was necessary to prepare UDP-GlcNAc and acetyl-CoA in gram quantities. We were able to produce such quantities using *in vitro* enzymatic approaches.

Specifically, for the production of UDP-GlcNAc, we modified a previously published procedure (9) and cloned and purified the *Escherichia coli* W3110 UTP-glucose-1-phosphate uridylyltransferase, the *E. coli* W3110 inorganic pyrophosphatase, and the *Bifidobacterium longum* NaHK kinase required for the *in vitro* synthesis. As outlined in Experimental procedures, we were able to prepare ∼10 g of UDP-GlcNAc (typical cost for commercially available UDP-GlcNAc is $600 for 500 mg).

The *in vitro* synthesis of acetyl-CoA was accomplished in two steps. The first step produced CoA starting from pantothenic acid. The following enzymes required for CoA biosynthesis, namely CoaA, CoaBC, CoaD, and CoaE, were cloned and purified to homogeneity from *E. coli* W3110. Following the *in vitro* synthesis of CoA and its subsequent purification, acetyl-CoA was produced using acetyl-CoA synthase, which was cloned from *E. coli* W3110 and purified in the laboratory. We produced ∼7 g of acetyl-CoA (typical cost for commercially available acetyl-CoA is between $700 and $1000 per 100 mg).

### Kinetics of the aminotransferase (*Pcryo*_0638)

**Figure 3:**
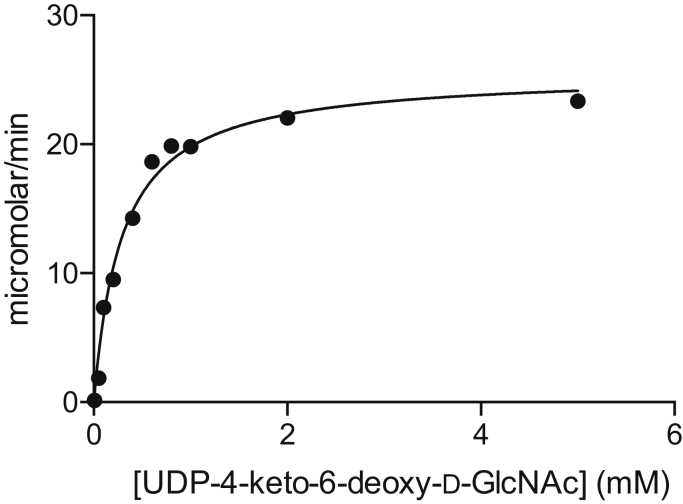
**Plot of initial velocities versus substrate concentration for *Pcryo*_0638.** In presenting the data as we do, we are adhering to standard conventions in enzymology. Measuring velocities over a wide range of substrate concentrations allows us to obtain data that define both *k*_cat_ and *k*_cat_/*K*_M_ well, which is not accomplished by measuring replicates at fewer different concentrations. The graph shown allows for a qualitative appreciation of the quality of the data; the quantitative goodness-of-fit to the Michaelis–Menten equation is given by the standard errors as described in Experimental procedures.

On the basis of DNA sequence analyses, we postulated that the 0638 gene encodes for an aminotransferase that functions on UDP-4-keto-6-deoxy-*N*-acetyl-d-glucosamine (Fig. 2). Shown in Figure 3 is a plot of the initial velocity kinetic data for *Pcryo*_0638 using a discontinuous HPLC-based assay. Relevant kinetic parameters are presented in Table 1. The overall catalytic efficiency of *Pcryo*_0638 is 6.6 x 10^3^ M^-1^s^-1^. This value is similar to those observed for other sugar aminotransferases presently being investigated in the laboratory, which lie in the range of 4–6 x 10^3^ M^-1^s^-1^ (to be published at a later date).

**Table 1:** Kinetic data for *Pcryo_0638* and *Pcryo_0637*

enzyme	Substrate	*K*_M_ (mM)	*k*_cat_ (s^-1^)	*k*_cat_/*K*_M_ (M^-1^s^-1^)
*Pcryo*_0638	UDP-4-keto-6-deoxy-*N*-acetyl-d-glucosamine	0.29 ± 0.04	1.9 ± 0.2	6.6 (±0.7) x 10^3^
*Pcryo*_0637	UDP-2-acetamido-4-amino-2,4,6-trideoxy-d-glucose	0.62 ± 0.05	545 ± 40	8.8 (±0.9) x 10^5^
*Pcryo*_0637	Acetyl-CoA	0.070 ± 0.008	1900 ± 200	2.7 (±0.3) x 10^7^

### Structure of the aminotransferase (*Pcryo*_0638)

Crystals of *Pcryo*_0638 were grown in the presence of PLP and UDP-2-acetamido-4-amino-2,4,6-trideoxy-d-glucose (the product). They belonged to the triclinic space group *P*1 with a complete dimer in the asymmetric unit. The structure was determined to 1.3 Å resolution, and the model was refined to an overall *R*-factor of 15.1% (relevant refinement statistics are provided in Table 2).

**Table 2:** X-ray data collection statistics and model refinement statistics

X-ray data and model parameters	*Pcryo*_0638	*Pcryo*_0637 UDP/acetyl-CoA	*Pcryo*_0637 CoA/UDP-2,4-diacetamido-2,4,6-trideoxy-d-glucose	*Pcryo*_0637 CoA/UDP-2-acetamido-4-amino-2,4,6-trideoxy-d-glucose	*Pcryo*_0637 tetrahedral intermediate
Resolution limits (Å)	50.0–1.30 (1.40–1.30)^a^	50.0–1.30 (1.40–1.30)^a^	50.0–1.55 (1.65–1.55)^a^	50.0–1.30 (1.40–1.30)^a^	50.0–1.40 (1.50–1.40)^a^
Number of independent reflections	202026 (36,011)	56,307 (11,060)	33,312 (5566)	56,136 (10,804)	45,470 (8481)
Completeness (%)	92.9 (83.0)	98.2 (96.6)	98.1 (95.0)	98.3 (94.6)	98.8 (97.9)
Redundancy	2.4 (1.9)	6.1 (2.8)	5.1 (2.4)	8.1 (2.5)	5.9 (3.3)
avg I/avg σ(I)	7.2 (1.9)	17.6 (3.0)	19.6 (3.0)	20.3 (2.4)	20.2 (3.3)
*R*_sym_ (%)^b^	5.7 (22.9)	6.2 (43.6)	4.5 (34.1)	5.4 (44.8)	4.9 (38.0)
c*R*-factor (overall)%/no. reflections	15.1/202026	19.5/56,307	16.4/33,312	17.7/56,136	15.9/45,470
*R*-factor (working)%/no. reflections	15.0/191942	19.4/53,390	16.2/31,600	17.3/53,279	15.8/43,279
*R*-factor (free)%/no. reflections	16.9/10,084	21.4/2917	19.0/1712	19.4/2857	18.2/2191
Number of protein atoms	6354	1642	1635	1649	1651
Number of heteroatoms	1480	364	355	373	379
Average B values					
Protein atoms (Å^2^)	12.2	11.8	17.8	14.7	14.5
Ligand (Å^2^)	10.2	18.6	34.1	24.6	23.4
Solvent (Å^2^)	28.1	26.6	33.5	29.5	30.1
Weighted RMS deviations from ideality					
Bond lengths (Å)	0.010	0.010	0.009	0.010	0.010
Bond angles (°)	1.55	1.90	1.60	1.60	1.70
Planar groups (Å)	0.009	0.008	0.008	0.008	0.009
Ramachandran regions (%)^d^					
Most favored	98.6	94.9	95.8	96.3	95.8
Additionally allowed	1.4	5.1	4.2	3.7	4.2
Generously allowed	0.0	0.0	0.0	0.0	0.0
PDB code	7L7X	7L7Y	7L7Z	7L81	7L82

a Statistics for the highest resolution bin.

b $R_{sym}=\left(\sum |\text{I}-\bar{\text{I}}|/\sum \text{I}\right)\times 100$.

c *R*-factor = (Σ|*F*_o_ - *F*_c_|/Σ|F_o_|) x 100 where *F*_o_ is the observed structure-factor amplitude and *F*_c_ is the calculated structure-factor amplitude.

d Distribution of Ramachandran angles according to PROCHECK (26).

**Figure 4:**
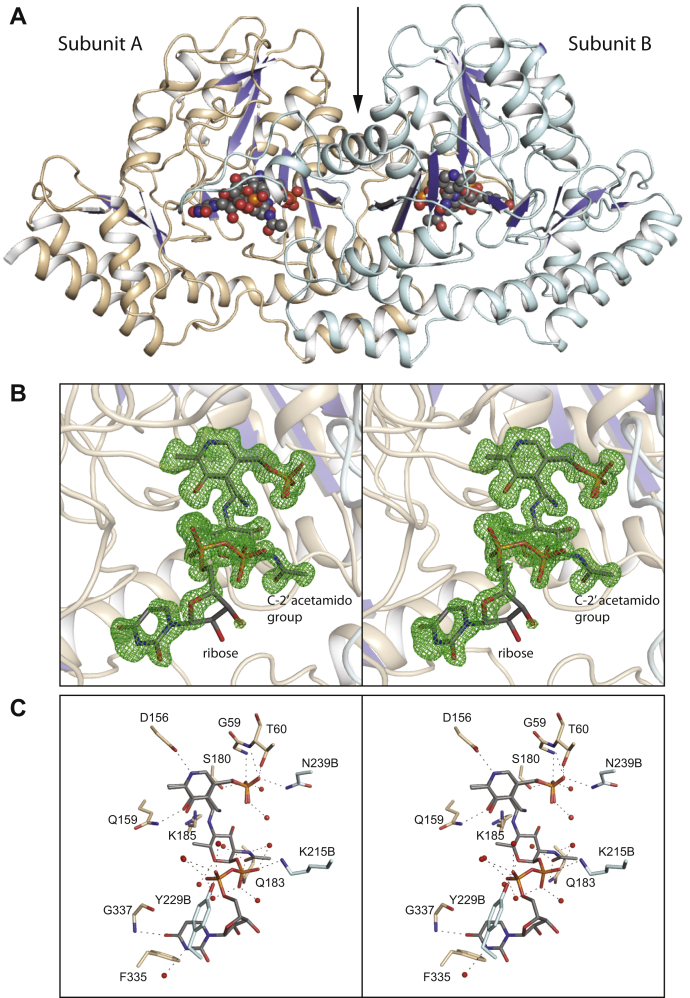
**The structure of *Pcryo*_0638. A ribbon representations of the dimer is shown in (*A*).** The *arrow* indicates the position of the twofold rotational axis that lies in the plane of the page. A close-up stereo view of the electron density corresponding to the bound external aldimine/PMP mixture is shown in (*B*). The map was calculated with (*F*_o_-*F*_c_) coefficients and contoured at 3σ. The ligand was not included in the X-ray coordinate file used to calculate the omit map, and thus there is no model bias. Shown in stereo in (*C*) is a close-up view of the active site. Ordered water molecules are represented as spheres. Potential hydrogen bonds (within 3.2 Å) are indicated by the *dashed lines*. Side chains highlighted in wheat belong to subunit A, whereas those colored in light blue are contributed by subunit B. This figure and Figure 6, Figure 7, Figure 8, and Figure 10, Figure 11, Figure 12 were prepared with PyMOL (27).

Shown in Figure 4*A* is a ribbon representation of the dimer, which has an extensive total buried surface area of ∼5000 Å^2^. The electron densities for the two polypeptide chains of the dimer were continuous from Ala 5 to Asn 399. The α-carbons for the two subunits superimpose with a root-mean-square deviation of 0.14 Å, and thus the following discussion will refer only to subunit A unless otherwise indicated.

Each subunit contains a seven-stranded mixed β-sheet, a three-stranded antiparallel β-sheet, a β-hairpin motif, and 12 α-helices (one of which contains 37 residues). There is a *cis*-peptide bond located between Trp 9 and Pro 10. The indole nitrogen of Trp 9 lies within 2.9 Å of the carbonyl group of Lys 185, the conserved lysine in aminotransferases that forms the Schiff base of the internal aldimine.

The observed electron density for the ligand is displayed in Figure 4*B*. Given that the crystals were grown in the presence of PLP and the product, initial inspection of the map suggested that the external aldimine was bound. Even after modeling the ligand into the map, however, there was residual electron density calculated with (*F*_o_–*F*_c_) coefficients. To account for this observation, a mixture of the external aldimine and PMP was subsequently built into the electron density with each given half-occupancy.

As can be seen, the electron density corresponding to the ribose sugar of the external aldimine is not well defined. This is most likely due to the lack of interactions between it and the protein as can be seen in Figure 4*C*. The uracil ring, however, is sandwiched between the side chains of Phe 335 and Tyr 229, which is contributed by subunit B. There are two additional interactions between it and the backbone amide of Gly 337 and an ordered water molecule. The side chains of Tyr 229 and Lys 215, both contributed by subunit B, lie within 3.2 Å of an α- and a β-phosphoryl oxygen, respectively. Seven water molecules also participate in hydrogen-bonding interactions with the phosphoryl oxygens. The pyranosyl moiety of the external aldimine adopts the ^4^C_1_ chair conformation. The C-2' acetamido group is anchored into the active site by the side chain of Gln 183 and a water molecule, whereas the pyranosyl ring oxygen lies at 3.0 Å from a water molecule. There are no interactions within 3.2 Å of the C-3' hydroxyl group and the protein. As is typical for PLP-dependent enzymes, the nitrogen and the oxygen of the pyridoxal moiety lie within 2.6 Å and 2.9 Å, respectively, to the side chains of Asp 156 and Gln 159.

### Kinetics of the *N*-acetyltransferase (*Pcryo*_0637)

**Figure 5:**
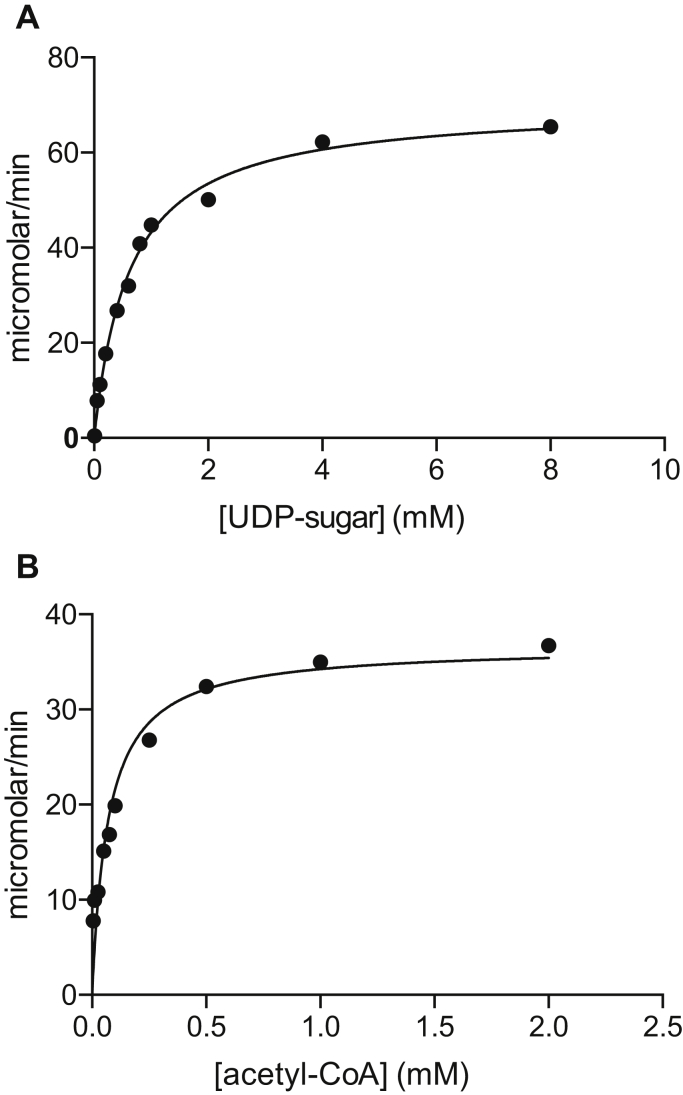
**Plot of initial velocities versus substrate concentrations for *Pcryo*_0637**.

Shown in Figure 5 are plots of the initial velocity kinetic data for *Pcryo*_0637 again using a discontinuous HPLC-based assay and varying either the concentrations of UDP-2-acetamido-4-amino-2,4,6-trideoxy-d-glucose or acetyl-CoA. Relevant kinetic parameters are provided in Table 1. The catalytic efficiency of the enzyme with the UDP-sugar substrate is 8.8 (±0.9) x 10^5^ M^-1^s^-1^. This value is similar to that observed for other *N*-acetyltransferases investigated in the laboratory (10, 11, 12, 13).

### Structure of the *N*-acetyltransferase (*Pcryo*_0637)

For the structural analysis of *Pcryo*_0637 described here, four different protein/ligand complexes were determined to high resolution.

**Figure 6:**
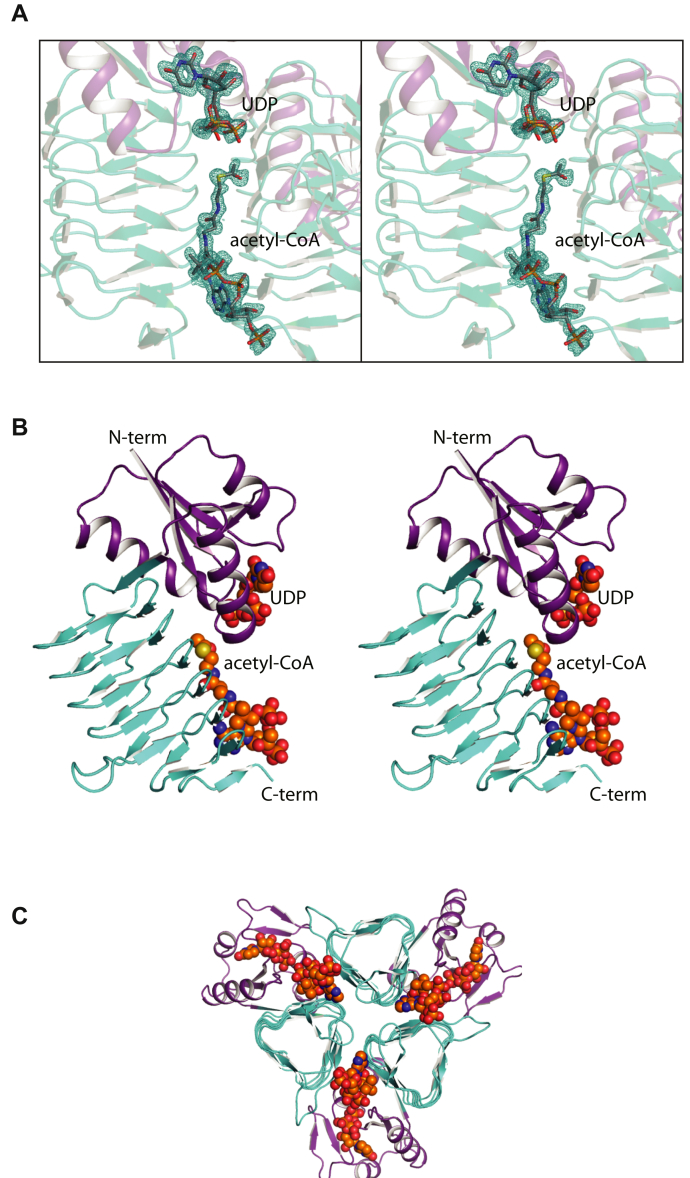
**Structure of the *Pcryo*_0637 UDP/acetyl-CoA complex.** Shown in stereo in (*A*) is the observed electron density for the ligands calculated with (*F*_o_-*F*_c_) coefficients. The map was contoured at 3σ. A ribbon representation of the subunit in the asymmetric unit is shown in stereo in (*B*). The ligands are displayed in sphere representations. A ribbon representation of the trimer is shown in (*C*) and as can be seen the active sites are wedged between the subunits.

The first model of *Pcryo*_0637 to be described, namely that with bound UDP and acetyl-CoA, was refined to an *R*-factor of 19.5% at 1.3 Å resolution (model statistics are presented in Table 2). The crystals utilized for the investigation belonged to the space group *R*3 with one subunit in the asymmetric unit. Shown in Figure 6*A* is the electron density corresponding to the bound ligands. As can be seen, the electron density for the bound acetyl-CoA is well defined, whereas in the case of the UDP moiety it appears that the pyrophosphoryl group adopts two alternate conformations.

The *N*-acetyltransferases belonging to the left-handed β-helix superfamily are known to function as trimers, and thus the local threefold rotational axis of *Pcryo*_0637 packed along a crystallographic triad. A ribbon representation of one subunit is provided in Figure 6*B*. The overall architecture of the subunit is distinctly bilobal with an N-terminal domain delineated from Ser 2 to Ser 93 and the C-terminal domain formed by Leu 94 to Ala 218. The N-terminal domain, characterized by a five-stranded mixed β-sheet surrounded by three α-helices, provides a binding platform for the UDP molecule. The C-terminal domain displays a classical left-handed β-helix motif with six complete turns. As can be seen, the acetyl-CoA ligand adopts an extended conformation, which allows the placement of the acetyl group into the active site region that is located at the interface of the two domains. A ribbon representation of the complete trimer is depicted in Figure 6*C*. The left-handed β-helices provide most of the subunit:subunit interactions. Indeed, whereas the total buried surface area for each subunit is ∼1850 Å^2^, 1200 Å^2^ arises from the left-handed β-helix motif. The acetyl-CoA moieties are wedged between the subunits of the trimer. There is one *cis*-peptide bond between Asn 212 and Pro 213. This *cis*-peptide is positioned in the last reverse turn of the subunit and from previous investigations is known to adopt the *trans*-conformation in the absence of CoA (14,15).

**Figure 7:**
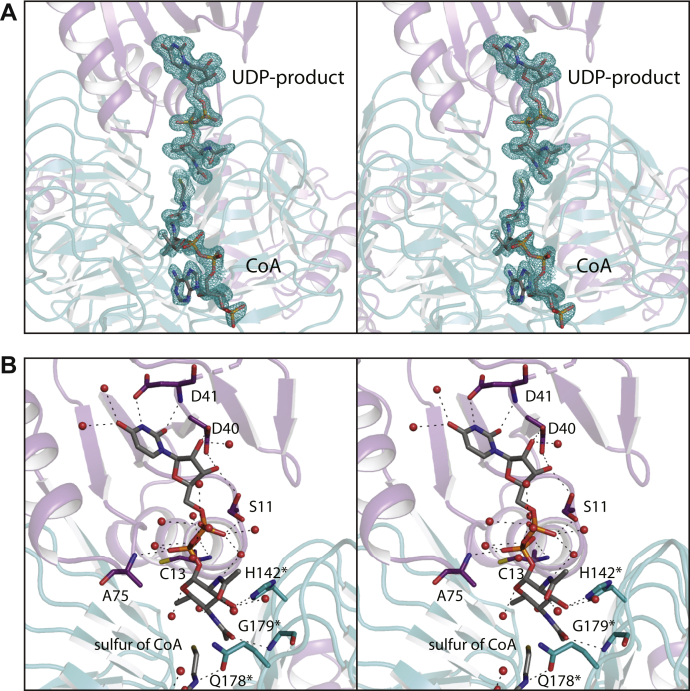
**Structure of the *Pcryo*_0637 UDP-sugar product/CoA complex.** The electron density for the ligands, calculated with (*F*_o_-*F*_c_) coefficients and contoured at 3σ, is shown in stereo in (*A*). A close-up view of the region surrounding the ligands is displayed in (*B*) in stereo. The *dashed lines* indicate possible hydrogen-bonding interactions (3.2 Å or less). Ordered water molecules are displayed as *red spheres*. Those residue labels marked by *asterisks* are contributed by a neighboring subunit.

The second model of *Pcryo*_0637 to be determined was that of a ternary complex with UDP-2,4-diacetamido-2,4,6-trideoxy-d-glucose (the product) and CoA. The model was refined to an *R*-factor of 16.4% at 1.55 Å resolution. As shown in Figure 7*A*, the electron density corresponding to the bound ligands is unambiguous. A close-up view of the active site surrounding the UDP-product is presented in Figure 7*B*. The uracil ring is anchored into the active site by the backbone amide and the carboxylate side chain of Asp 41 and two ordered water molecules. The side chains of Asp 40 and Ser 11, along with a water molecule, lie within 3.2 Å of the ribose hydroxyl groups. Note that the side chain of Ser 11 adopts two conformations, the second of which allows it to form a hydrogen bond with an α-phosphoryl oxygen of the UDP-sugar product. Additionally, the phosphoryl oxygens are surrounded by numerous water molecules and the backbone amide groups of Cys 13 and Ala 75. The pyranosyl group of the UDP-sugar product directly interacts only with the side chain of His 142 and the backbone amide group of Gly 179 (both from a neighboring subunit of the trimer). On the basis of previously determined structures of related *N*-acetyltransferases, His 142 most likely functions as the active site base (14,15).

**Figure 8:**
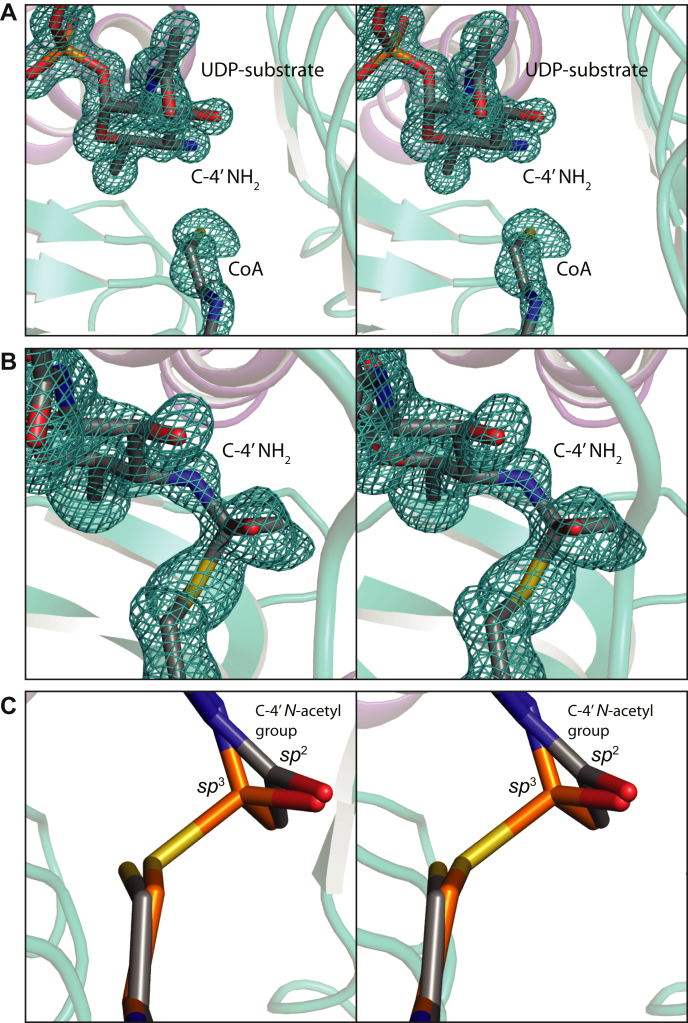
**Structure of the *Pcryo*_0637 UDP-sugar substrate/CoA complex.** The electron density for the ligands, calculated with (*F*_o_-*F*_c_) coefficients and contoured at 3σ, is shown in stereo in (*A*). Interestingly, when the *Pcryo*_0637 crystals were soaked in acetyl-CoA and the UDP-sugar substrate, the electron density shown in (*B*) was observed. The map was calculated with (*F*_o_-*F*_c_) coefficients and contoured at 3σ. Clearly a tetrahedral intermediate was trapped. The superposition of the two complexes near the sulfur of CoA, as presented in (*C*), demonstrates the movement of the atoms in collapsing from *sp*^3^ to *sp*^2^ hybridization.

To address possible side chain movements in the active site upon catalysis, the third model of *Pcryo*_0637 to be solved was that of a ternary complex with UDP-2-acetamido-4-amino-2,4,6-trideoxy-d-glucose (the substrate) and CoA. The model was refined at 1.3 Å resolution to an overall *R*-factor of 17.7%. Shown in Figure 8*A* is the electron density for the corresponding ligands. Again, the conformations of the UDP-sugar substrate and CoA in the active site are unambiguous. The α-carbons for the two models, with either bound UDP-sugar substrate or UDP product, superimpose with a root-mean-square deviation of 0.08 Å, and there are no movements of the side chains in the active site cleft within experimental error.

For the last structural analysis, the crystals of *Pcryo*_0637 were soaked in acetyl-CoA and the substrate, UDP-2-acetamido-4-amino-2,4,6-trideoxy-d-glucose in an attempt to visualize the Michaelis complex. It was anticipated that either the active site pocket would contain acetyl-CoA and the UDP-sugar substrate, or, if the enzyme was active in the crystalline lattice, it would show bound CoA and the UDP-sugar product. Unexpectedly, as can be seen from the electron density provided in Figure 8*B*, the tetrahedral intermediate was, in fact, trapped. The oxygen of the intermediate lies within 3.1 Å of the backbone amide group of Gly 160 from a neighboring subunit, thus providing an oxyanion hole.

A superposition of the coordinates for this tetrahedral intermediate onto those of the enzyme/UDP-product/CoA complex reveals a movement of ∼0.6 Å in transitioning from *sp*^*3*^ to *sp*^*2*^ hybridization (Fig. 8*C*). The factors stabilizing the tetrahedral intermediate are unclear, but the electron density is unambiguous. This trapping of a tetrahedral intermediate was previously reported in the structure analysis of perosamine *N*-acetyltransferase from our laboratory (13).

## Discussion

### Comparison of *Pcryo*_0638 to PglE and DesI

**Figure 9:**
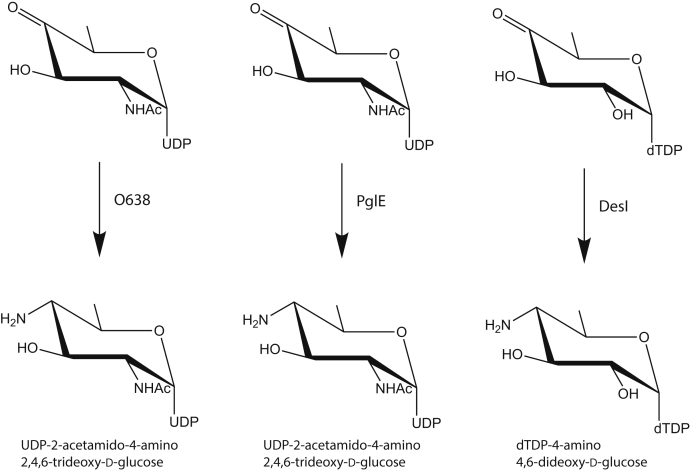
**Comparison of the reactions catalyzed by *Pcyro*_0638, PglE, and Desl**.

Shown in Figure 9 are the reactions catalyzed by PglE from *C*. *jejuni* and DesI from *Streptomyces venezuelae* (16,17). The substrate for PglE is identical to that for *Pcryo*_0638, whereas the substrate for DesI differs by having a hydroxyl group at C-2' and using dTDP as the nucleotide handle.

**Figure 10:**
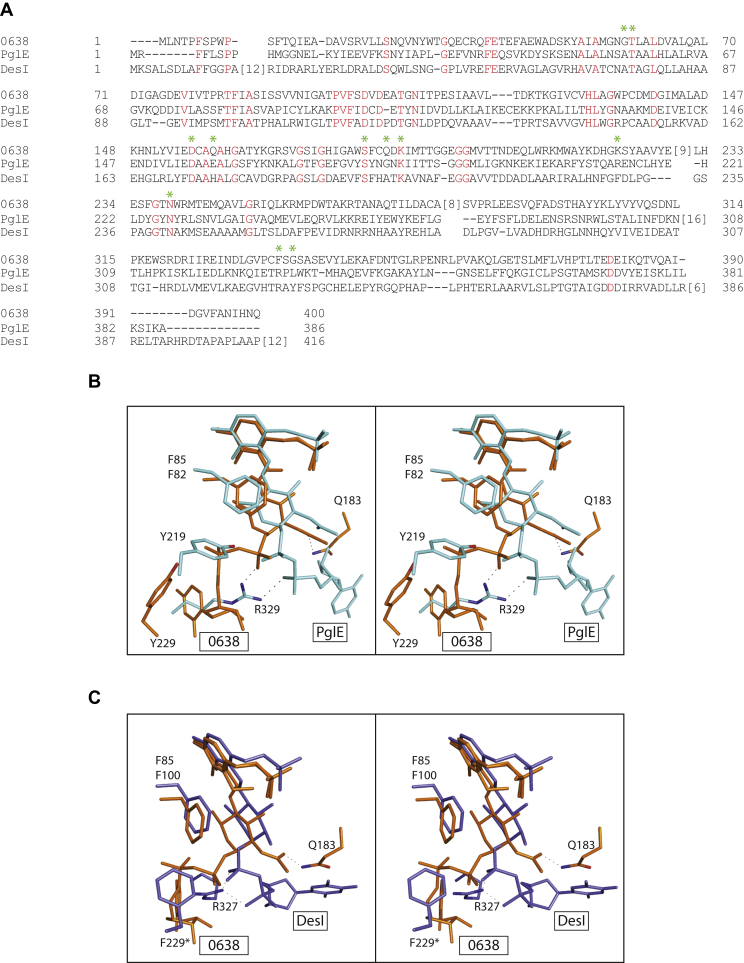
**Comparison of *Pcryo*_0638 to PglE and DesI.** An amino acid sequence alignment of these three aminotransferases is presented in (*A*). A superposition of *Pcryo*_0638 and PglE near the active site is displayed in stereo in (*B*). *Pcryo*_0638 and PglE are highlighted in *orange* and *light blue* bonds, respectively. Shown in (*C*) is a superposition of *Pcryo*_0638 and DesI colored in *orange* and *blue* bonds, respectively.

An amino acid sequence alignment for these three proteins is presented in Figure 10*A*. The residues marked by the asterisks are those located within hydrogen-bonding distance of the external aldimine in *Pcryo*_0638. The only residues that are strictly conserved among these three proteins are Thr 60, Asp 156, Ser 180, Lys 185, and Asn 239 (*Pcryo*_0638 numbering).

Shown in Figure 10*B* is a superposition of the observed positions for the external aldimines in *Pcryo*_0638 and PglE. The α-carbons for these two proteins correspond with a root-mean-square deviation of 2.3 Å. With respect to amino acid sequence, the two enzymes demonstrate a 27% identity and a 50% similarity. Whereas both enzymes function on the same substrate, the manner in which the ligands are accommodated in the active sites is remarkably different. In *Pcryo*_0638, the carboxamide group of Gln 183 interacts via a hydrogen bond with the C-2' acetamido group of the sugar. In PglE, the structurally equivalent residue is a glycine. Indeed, the position of Gln 183 precludes the ligand from binding to *Pcryo*_0638 in the manner observed for PglE. On the basis of the amino acid sequence, it might have been postulated that Tyr 229 and Tyr 219 in *Pcryo*_0638 and PglE, respectively, would adopt similar orientations in the active site. As can be seen, however, the orientation of Tyr 219 in PglE precludes the ligand from binding in the manner observed for *Pcryo*_0638.

DesI and *Pcryo*_0638 show an amino acid sequence identity and similarity of 34% and 48%, respectively and their α-carbon positions superimpose with a root-mean-square deviation of 2.5 Å. The difference in ligand binding between *Pcryo*_0638 and DesI is equally fascinating as can be seen in Figure 10*C*. In DesI, the aromatic ring of Phe 229 (subunit B) and the guanidinium group of Arg 372 are the major driving forces for the alternate ligand-binding conformation. Indeed, in *Pcryo*_0638, these residues are a lysine and a phenylalanine, respectively.

The striking difference in ligand binding between these three proteins yet again emphasizes the perils of model building and/or assumptions made simply on amino acid sequence alignments.

### Comparison of *Pcryo*_0637 to PglD

**Figure 11:**
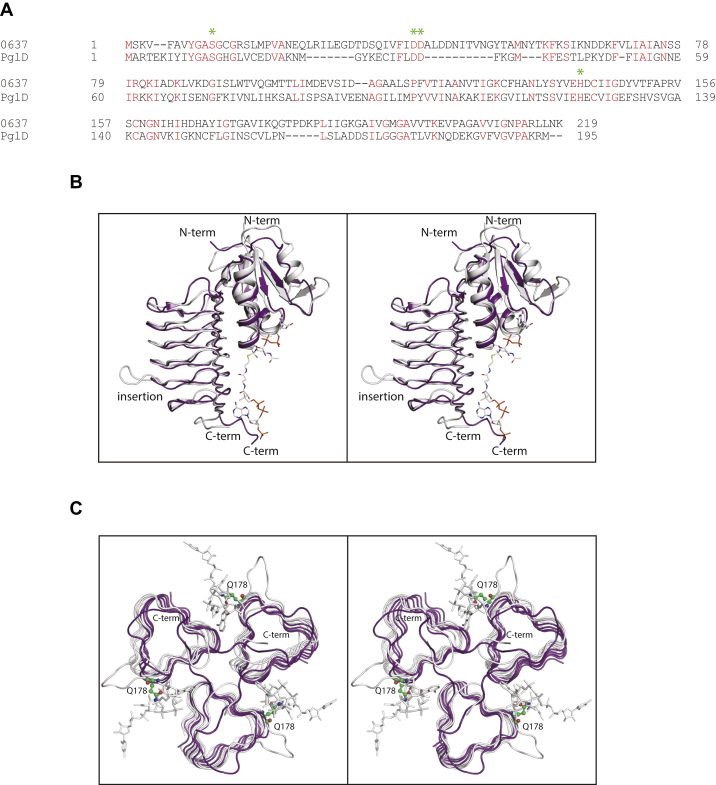
**Comparison of *Pcryo*_0637 to PglD.** An amino acid sequence alignment between these two *N*-acetyltransferases is provided in (*A*). A superposition of the ribbon representations for the *Pcryo*_0637 (tetrahedral intermediate) and the apo-PglD subunits is shown in stereo in (*B*). They are colored in *white* and *violet*, respectively, and the tetrahedral intermediate in *Pcryo*_0637 is drawn in a stick representation. There is only one major insertion in *Pcryo*_0637 relative to PglD. A superposition of the trimeric forms of these enzymes is provided in stereo in (*C*). Note the domain swapping that occurs in apo-PglD (*violet*). The side chain of Gln 178 in *Pcryo*_0637 is drawn in a ball-and-stick representation and colored in *green*.

The first biochemical characterization of an *N*-acetyltransferase that functions on UDP-2-acetamido-4-amino-2,4,6-trideoxy-d-glucose, namely PglD from *C*. *jejuni*, was reported in 2006 (18). Structural analyses of PglD were subsequently published by two independent research groups (14,15). Shown in Figure 11*A* is an amino acid sequence alignment between *Pcryo*_0637 and PglD. These two enzymes demonstrate 28% sequence identities and 45% similarities, and their α-carbon positions correspond with a root-mean-square deviation of 1.3 Å. Shown in Figure 11*B* is a superposition of the ribbon drawings for these two proteins using the coordinates for the tetrahedral intermediate of *Pcryo*_0637 determined in this investigation and the apo-form of PglD determined by Rangarajan *et al.,* (14). There is only one insertion in *Pcryo*_0637 that lies between Gly 179 and Leu 185. This region, which splays away from the main body of the trimer, allows Gln 178 of *Pcryo*_0637 to be positioned into the active site cleft as can be seen in Figure 11*C*. As noted above, there is a *trans* to *cis* conformational change of a conserved proline (Pro 213 in *Pcryo*_0637) that occurs upon CoA binding. In the absence of CoA, domain swapping occurs with the proline in the *trans*-conformation, whereas in the presence of the cofactor, the random coil (defined by *cis*-Pro 213 to Leu 217 in *Pcryo*_0637) loops back to its original subunit to participate in the last β-strand of the β-helix (Fig. 11*C*).

**Figure 12:**
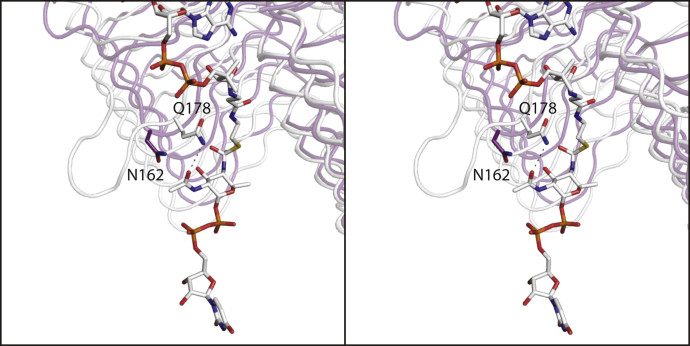
**Comparison of *Pcryo*_0637 to PglD near the C-2' acetamido moiety of the UDP-sugar ligand.** Due to the insertion indicated in Figure 11, *Pcryo*_0637 (*white*) and PglD (*violet*) differ with respect to their interactions with the C-2' acetamido group of the substrate.

Most of the residues involved in UDP-sugar binding (Fig. 7*B*) are conserved in PglD. These include Ser 11, Asp 40, Asp 41, and His 142 (Ser 13, Asp 35, Asp 36, and His 125 in PglD.) There are two differences, however. Cys 13 in *Pcryo*_0637, which abuts one side of the pyranosyl group of the ligand, is His 15 in PglD. Also, Gln 178, which participates in a hydrogen-bonding interaction with the sugar C-2' acetamido group, is Asn 162 in PglD. A close-up view of this difference is presented in Figure 12.

In summary, we have demonstrated that the genes in *P. cryohalolentis,* namely 0638 and 0637, encode for a PLP-dependent aminotransferase and an *N*-acetyltransferase, respectively. Whereas the number of different carbohydrate residues in higher organisms is limited to about 13, more than a hundred different di-, tri-, and tetradeoxysugars have been isolated from prokaryotic sources thus far, and the list continues to grow on a yearly basis. Indeed, the diversity of prokaryotic carbohydrate structures observed in the natural world is extraordinary. In the coming years it is anticipated that each biochemical study of the enzymes involved in their biosynthesis will yield new stories with some unexpected twists and turns as in the case of *Pyro*_0638 and *Pyro*_0637.

## Experimental procedures

### Protein expression and purification

The genes encoding *Pcryo*_0637 and *Pcryo*_0638 from *P. cryohalolentis* K5^T^ (ATCC BAA-1226) were cloned using Platinum Pfx DNA polymerase (Invitrogen). The *Pcryo*_0637 gene was placed into a pET31(b+) plasmid (Novagen) to generate a protein with a noncleavable C-terminal polyhistidine tag (LEHHHHHH) for crystallization. The *Pcryo*_0638 gene was placed into a pET28t3g plasmid, a modified pET28b(+) vector (Novagen), which leads to a protein with an N-terminal polyhistidine tag possessing an rTEV cleavage site as previously described (19). For measurement of the *Pcryo*_0637 kinetic properties, the gene encoding it was also placed into the pET28t3g plasmid to allow removal of the histidine tag. The expression plasmids were utilized to transform Rosetta2(DE3) *E*. *coli* cells (Novagen) for protein expression. Cultures were grown in lysogeny broth supplemented with 100 mg/L ampicillin and 50 mg/L chloramphenicol for pET31-*Pcryo_*0637 or 50 mg/L kanamycin and 50 mg/L chloramphenicol for pET28t3g-*Pcryo_*0638 and pET28t3g-*Pcryo_*0637. The cultures were grown at 37 °C with shaking until an optical density of 0.8 was reached at 600 nm. The flasks were cooled in an ice bath, and the cells were induced with 1 mM isopropyl β-d-1-thiogalactopyranoside and allowed to express protein at 21 °C for 24 h.

The cells were harvested by centrifugation and frozen as pellets in liquid nitrogen. These pellets were subsequently disrupted by sonication on ice in a lysis buffer composed of 50 mM sodium phosphate, 20 mM imidazole, 10% glycerol, and 300 mM NaCl (pH 8.0). The lysates were cleared by centrifugation, and the proteins were purified at 4 ^°^C utilizing Prometheus Ni-NTA agarose (Prometheus Protein Biology Products) according to the manufacturer’s instructions. All buffers were adjusted to pH 8.0 and contained 50 mM sodium phosphate, 300 mM NaCl, and imidazole concentrations of 20 mM for the wash buffer and 300 mM for the elution buffer. C-terminally tagged *Pcryo*_0637 was dialyzed against 10 mM Tris-HCl (pH 8.0) and 200 mM NaCl and then concentrated to 27.5 mg/ml based on an extinction coefficient of 1.37 (mg/ml)^−1^ cm^−1^. The polyhistidine tags were removed from *Pcryo*_0638 and *Pcryo*_0637 by digestion with rTEV protease. The rTEV protease and remaining tagged proteins were removed by passage over Ni-NTA agarose, and the tag-free proteins were dialyzed against 10 mM Tris-HCl (pH 8.0) and 200 mM NaCl. The *Pcryo*_0638 was concentrated to 25 mg/ml based on an extinction coefficient of 0.51 (mg/ml)^-1^ cm^-1^. The tag-free *Pcryo*_0637 was concentrated to 28 mg/ml.

### Crystallization

Crystals of *Pcryo*_0638 were grown *via* the hanging drop method of vapor diffusion at room temperature from 18 to 22% poly(ethylene glycol) 8000, 200 mM tetramethylammonium chloride, and 100 mM HEPPS (pH 8.0) in the presence of 1 mM PLP and 5 mM UDP-2-acetamido-4-amino-2,4,6-trideoxy-d-glucose. They belonged to the triclinic space group *P*1 with unit cell dimensions of *a* = 57.5 Å, *b* = 63.1 Å, *c* = 69.9 Å, α = 81.8^o^, β = 78.6^o^, and γ = 66.8^o^. The asymmetric unit contained one dimer. For X-ray data collection, the crystals were transferred to a cryo-protectant solution composed of 26% poly(ethylene glycol) 8000, 250 mM NaCl, 250 mM tetramethylammonium chloride, 14% ethylene glycol, 1 mM PLP, 5 mM UDP-2-acetamido-4-amino-2,4,6-trideoxy-d-glucose, and 100 mM HEPPS (pH 8.0).

Crystals of *Pcryo*_0637 were grown *via* the hanging drop method of vapor diffusion at room temperature from 23 to 26% 2-methyl-2,4-pentanediol buffered with 100 mM HEPES (pH 7.5) in the presence of 5 mM CoA and 5 mM UDP-2-acetamido-4-amino-2,4,6,-trideoxy-d-glucose. They belonged to the rhombohedral space group *R*3 with unit cell dimensions of *a* = *b* = 97.1 Å and *c* = 60.0 Å The asymmetric unit contained one subunit. For X-ray data collection, the crystals were transferred to a cryo-protectant solution composed of 40% 2-methyl-2,4-pentanediol, 200 mM NaCl, 5% ethylene glycol, 5 mM CoA, 5 mM UDP-2-acetamido-4-amino-2,4,6,-trideoxy-d-glucose, and 100 mM HEPES (pH 7.5). Crystals of the protein/UDP/acetyl-CoA complex were grown under the same conditions using 5 mM UDP and 5 mM acetyl-CoA. For the protein/CoA/UDP-2,4-diacetamido-2,4,6-trideoxy-d-glucose complex, crystals were grown in the presence of 5 mM CoA and 20 mM UDP-2,4-diacetamido-2,4,6-trideoxy-d-glucose. The intermediate was obtained by first taking crystals grown in the presence of UDP and acetyl-CoA and soaking them in 30% 2-methyl-2,4-pentanediol, 200 mM NaCl, 100 mM HEPES (pH 7.5), and 1 mM acetyl-CoA to remove excess UDP. They were then transferred to a soaking solution that contained 32% 2-methyl-2,4-pentanediol, 200 mM NaCl, 100 mM HEPES (pH 7.5), 5 mM acetyl-CoA, and 5 mM UDP-2-acetamido-4-amino-2,4,6-dideoxy-d-glucose.

The soaking solution was exchanged several times over 2 hours, and the crystals were then allowed to soak overnight.

### X-ray data collection and processing

All X-ray data were collected using a BRUKER D8-VENTURE sealed tube system equipped with HELIOS optics and a PHOTON II detector. The X-ray data sets were processed with SAINT and scaled with SADABS (Bruker AXS). Relevant X-ray data collection statistics are listed in Table 2.

### Structure solution and model refinement

*Pcryo*_0638 was solved *via* molecular replacement with Phaser (20) using PDB entry 3FRK (21) as a search probe. Iterative cycles of model building with COOT (22,23) and refinement with REFMAC (24) led to a final X-ray model with an overall *R*-factor of 15.1%. Relevant refinement statistics are provided in Table 2.

The *Pcryo*_0637/CoA/UDP-2-acetamido-4-amino-2,4,6-trideoxy-d-glucose complex model was solved *via* molecular replacement with Phaser (20) using PDB entry 4M9C (25) as a search probe. Iterative cycles of model building with COOT (22,23) and refinement with REFMAC (24) led to a final X-ray model with an overall *R*-factor of 17.7%. The remaining *Pcryo*_0637 complexes were solved *via* difference Fourier methods or molecular replacement as needed (depending upon the unit cell indexing) starting with the *Pcryo*_0637/CoA/UDP-2-acetamido-4-amino-2,4,6-trideoxy-d-glucose complex model. Relevant refinement statistics are provided in Table 2.

### Synthesis of UDP-*N*-acetyl-d-glucosamine (UDP-GlcNAc)

To circumvent the prohibitive costs of UDP-GlcNAc, we synthesized it in the laboratory *via* a modification of a published procedure (9). Specifically, we cloned and purified the *E. coli* W3110 UTP-glucose-1-phosphate uridylyltransferase, the *E. coli* W3110 inorganic pyrophosphatase, and the *B*. *longum* NaHK *N*-acetylhexosamine 1-kinase.

After purification of these three enzymes, a 100 ml solution containing 50 mM UTP, 50 mM Mg^2+^ATP, 60 mM HEPPS, and 60 mM GlcNAc was adjusted to pH 8.5. Solid MgCl_2_^.^6H_2_O was added to a final concentration of 200 mM. The inorganic pyrophosphatase (0.5 mg/ml final concentration), the *N*-acetylhexosamine 1-kinase (1 mg/ml final concentration), and UTP-glucose-1-phosphate uridylyltransferase (1.5 mg/ml final concentration) were subsequently added. The enzymatic synthesis was allowed to proceed overnight at 37^°^C. Precipitates were removed, followed by removal of enzymes by passage through a 30 kDa filtration membrane. The filtrate was diluted with water, adjusted to pH 4.0, and loaded onto a 600 ml fast flow Q-sepharose column. Reaction products were separated with a 4 L linear gradient (0–2 M ammonium acetate, pH 4.0). Purified UDP-GlcNAc was pooled and lyophilized.

### Synthesis of UDP-4-keto-6-deoxy-*N*-acetyl-d-glucosamine

A buffered solution of 50 mM UDP-GlcNAc was prepared in 50 mM HEPPS (pH 8.0). To this solution, 20 mg/ml of PglF, a UDP-*N*-acetyl-glucosamine 4,6-dehydratase from *C. jejuni*, was added (8). The reaction was allowed to proceed overnight at room temperature. After removal of the enzyme by filtration through a 30 kDa membrane, the conversion of UDP-GlcNAc to the 4-keto sugar product was verified by HPLC.

### Synthesis of UDP-2-acetamido-4-amino-2,4,6-trideoxy-d-glucose

UDP-2-acetamido-4-amino-2,4,6-trideoxy-d-glucose was made on a large scale starting with 1.5 g UDP-GlcNAc. The 450 ml reaction mixtures contained 5.5 mM UDP-GlcNAc, 50 mM sodium glutamate, 0.2 mM PLP, 0.5 mg/ml PglF, and 1 mg/ml PglE (an aminotransferase from *C. jejuni*) (16). The reaction was allowed to proceed overnight at room temperature. Enzymes were removed by filtration through a 30 kDa membrane. The UDP-2-acetamido-4-amino-2,4,6-trideoxy-d-glucose was then purified in two steps. The filtered reaction mixture was diluted, adjusted to pH 4.0, and loaded onto a 600 ml DEAE Sepharose column. After washing with water, the UDP-2-acetamido-4-amino-2,4,6-trideoxy-d-glucose was eluted using a 3 L gradient (0–1.5 M) of ammonium acetate (pH 4.0). Following lyophilization, the UDP-2-acetamido-4-amino-2,4,6-trideoxy-d-glucose was then dissolved in water, the pH was adjusted to 8.5, and the solution was loaded onto a 50 ml HiLoad 26/10 Q Sepharose HP column and eluted with a 13 column volume gradient (0–0.8 M) ammonium bicarbonate (pH 8.5). Purified UDP-2-acetamido-4-amino-2,4,6-trideoxy-d-glucose was pooled and lyophilized.

### Synthesis of UDP-2,4-diacetamido-2,4,6-trideoxy-d-glucose

In total, 200 ml reactions containing 50 mM HEPPS (pH 8.0), 3 mM UDP-2-acetamido-4-amino-2,4,6-trideoxy-d-glucose, 3.3 mM acetyl-CoA, and 0.5 mg/ml *Pcryo*_0637 were set up. The reactions were typically complete in 4 hours. After removal of the enzyme by filtration through a 30 kDa membrane, the filtrate was diluted with water and loaded onto a 50 ml HiLoad 26/10 Q Sepharose HP column, and the UDP-2,4-diactamido-2,4,6-trideoxy-d-glucose was purified using a 15 column volume gradient (0–0.8 M) ammonium bicarbonate (pH 8.5). Purified UDP-2,4-diacetamido-2,4,6-trideoxy-d-glucose was pooled and lyophilized.

### Synthesis of acetyl-CoA

Sizable amounts of acetyl-CoA were required for the large-scale syntheses of UDP-2,4-diacetamido-2,4,6-trideoxy-d-glucose and other related sugars required for this investigation. On our limited budget, we simply could not afford to purchase commercially available acetyl-CoA. As such, the required acetyl-CoA was synthesized *in vitro*. *E. coli* W3110 (ATCC 39936) possesses five enzymes that convert pantothenic acid into acetyl-CoA: CoaA, CoaBC, CoaD, CoaE, and acetyl-CoA synthase. The genes encoding these proteins were cloned and placed into expression vectors. With the exception of CoaA, which was placed into pET28t3g to generate an N-terminally tagged protein, the remainder were placed into pET31(b+) to give C-terminally tagged proteins. Cultures were grown in lysogeny broth supplemented with appropriate antibiotics at 37°C with shaking until an optical density of 0.8 was reached at 600 nm. The flasks were cooled in an ice bath, and the cells were induced with 1 mM isopropyl β-d-1-thiogalactopyranoside and allowed to express protein at 21°C for 24 h.

The cells were harvested by centrifugation and frozen as pellets in liquid nitrogen, with the exception of CoaBC, which was purified immediately upon harvesting. CoaA, CoaD, CoaE, and acetyl-CoA synthase were purified at 4^°^C as described above and dialyzed against 10 mM Tris-HCl (pH 8.0) and 200 mM NaCl. CoaBC was purified in an analogous manner but at room temperature, and the dialysis buffer was supplemented with 2 mM DTT. Proteins were concentrated as follows: CoaA to 15 mg/ml based on an extinction coefficient of 0.80 (mg/ml)^-1^ cm^-1^, CoaBC to 15 mg/ml based on an extinction coefficient of 1.54 (mg/ml)^-1^ cm^-1^, CoaD to 40 mg/ml based on an extinction coefficient of 2.08 (mg/ml)^-1^ cm^-1^, CoaE to 25 mg/ml based on an extinction coefficient of 1.33 (mg/ml)^-1^ cm^-1^, and acetyl-CoA synthase to 20 mg/ml based on an extinction coefficient of 0.52 (mg/ml)^-1^ cm^-1^.

Acetyl-CoA was synthesized in two steps. The first step involved the synthesis of CoA. A typical 400 ml reaction contained 5 mM calcium pantothenate, 10 mM CTP, 35 mM Mg^2+^ATP, 10 mM cysteine, and 50 mM HEPPS, which was then adjusted to pH 8.5. Magnesium chloride was added to a final concentration of 100 mM, followed by the additions of the CoaA, CoaBC, CoaD, and CoaE enzymes to a final concentration of 1 mg/ml each. The reaction was allowed to proceed overnight at room temperature. Precipitate was removed by centrifugation, and the enzymes were removed by filtration through a 30 kDa membrane. The filtrate was diluted with 0.6 M ammonium acetate (pH 4.0) and loaded onto a 600 ml fast flow Q-sepharose column. After washing with 0.6 M ammonium acetate, the CoA was eluted with a 5 L gradient (0.6–2.4 M) ammonium acetate. Purified CoA was pooled and lyophilized. It was then converted to acetyl-CoA using acetyl-CoA synthase. A typical 400 ml reaction contained 10 mM CoA, 15 mM Mg^+2^ATP, and 50 mM HEPPS. After adjusting the pH to 8.5, magnesium chloride was added to a final concentration of 30 mM. DTT was added to a final concentration of 1 mM, and the mixture was allowed to incubate at room temperature for 2 h. Acetyl-CoA synthase was then added to a final concentration of 1 mg/ml. The reaction proceeded overnight at room temperature after which precipitates were removed by centrifugation and enzyme removal by passage through a 50 kDa membrane. The filtrate was diluted with water, and the acetyl-CoA was purified using a 50 ml HiLoad 26/10 Q Sepharose HP column and eluted with a 20 column volume gradient (0–2.25 M) ammonium acetate (pH 4.0). Purified acetyl-CoA was pooled and lyophilized.

A typical reaction would start with 1.2 g of calcium pantothenate (0.005 mol pantothenate) and would yield approximately 3 g of CoA for a yield of ∼80%. The conversion of CoA to acetyl-CoA was 100%, although during lyophilization ∼5% of acetyl-CoA would decompose to CoA. Dephospho-CoA and dephospho-acetyl-CoA can be made in the same manner by not including CoaE in the reaction. Purification of the dephospho products is identical to that of the phosphorylated compounds with comparable yields.

### Determination of kinetic constants

Steady-state kinetic parameters for *Pcryo*_0638 were evaluated *via* a discontinuous assay using an ÄKTA HPLC system. Reaction rates were determined by calculating the amount of aminated product (UDP-2-acetamido-4-amino-2,4,6-trideoxy-d-glucose) formed on the basis of the peak area on an HPLC trace measured at 262 nm. The area was correlated to concentration *via* a calibration curve created with standard samples that had been treated in the same manner as the reaction aliquots. The 2 ml reactions contained 50 mM HEPPS (pH 8.0), 0.5 mM PLP, 40 mM sodium glutamate, and 0.01 mg/ml (0.2 μM) *Pcryo*_0638. The UDP-4-keto-6-deoxy-*N*-acetyl-d-glucosamine concentrations were varied from 0.01 to 5.0 mM. Nine 200 μl aliquots were taken over a 5 min time period and then quenched by the addition of 6 μl of 6 M HCl. Subsequently, 200 μl of carbon tetrachloride was then added, the samples were vortexed and spun at 14000 x g for 2 min. In total, 150 μl of the aqueous phase was taken for HPLC analysis. The samples were diluted with 2 ml of water and loaded onto a 1 ml ResQ column. The amino-containing sugar product was quantified after elution with a 12-column volume gradient from 0 to 1 M ammonium acetate (pH 4.0).

Steady-state kinetic parameters for *Pcryo*_0637 were also determined *via* a discontinuous assay using an ÄKTA HPLC system, this time monitoring the amount of acetylated product (UDP-2,4-diacetamido-2,4,6-trideoxy-d-glucose) formed. For reactions to determine binding parameters for UDP-2-acetamido-4-amino-2,4,6-trideoxy-d-glucose, the 2 ml reactions contained 50 mM HEPPS (pH 8.0), 1.5 mM acetyl-CoA, and 0.00005 mg/ml (2.1 nM) *Pcryo*_0637. The UDP-2-acetamido-4-amino-2,4,6-trideoxy-d-glucose concentration was varied from 0.01 to 8 mM. For reactions to determine binding parameters for acetyl-CoA, the 2 ml reactions contained 50 mM HEPPS (pH 8.0), 8 mM UDP-2-acetamido-4-amino-2,4,6-trideoxy-d-glucose, and 0.0000075 mg/ml (0.32 nM) *Pcryo*_0637. The acetyl-CoA concentration was varied from 0.005 to 2 mM. Samples were taken and quenched as described above, diluted with 2 ml water, and the UDP-2,4-diacetamido-2,4,6-trideoxy-d-glucose product was quantified after elution from a 1 ml ResQ column with a 15-column volume gradient from 0 to 3 M ammonium acetate (pH 4.0).

The data were fit to the equation: *v*_0_ = (*V*_max_[S])/(*K*_M_ + [S]). The *k*_cat_ values were calculated according to the equation: *k*_cat_ = *V*_max_/[E_T_]. Relative kinetic parameters can be found in Table 1.

## Data availability

X-ray coordinates and structure factors have been deposited in the Protein Data Bank under accession codes: 7L7X, 7L7Y, 7L7Z, 7L81, and 7L82.

## Conflict of interest

The authors declare that they have no conflicts of interest with the contents of this article.
